# Phytochemical Analysis, Pharmacological and Safety Evaluations of Halophytic Plant, *Salsola cyclophylla*

**DOI:** 10.3390/molecules26082384

**Published:** 2021-04-20

**Authors:** Hamdoon A. Mohammed, Mohsen S. Al-Omar, Salman A. A. Mohammed, Ahmad H. Alhowail, Hussein M. Eldeeb, Mohammed S. M. Sajid, Essam M. Abd-Elmoniem, Osama A. Alghulayqeh, Yasser I. Kandil, Riaz A. Khan

**Affiliations:** 1 Department of Medicinal Chemistry and Pharmacognosy, College of Pharmacy, Qassim University, Qassim 51452, Saudi Arabia; m.omar@qu.edu.sa; 2 Department of Pharmacognosy, Faculty of Pharmacy, Al-Azhar University, Cairo 11884, Egypt; 3 Medicinal Chemistry and Pharmacognosy Department, Faculty of Pharmacy, JUST, Irbid 22110, Jordan; 4 Department of Pharmacology and Toxicology, College of Pharmacy, Qassim University, Qassim 51452, Saudi Arabia; m.azmi@qu.edu.sa (S.A.A.M.); aalhowail@qu.edu.sa (A.H.A.); hu.ali@qu.edu.sa (H.M.E.); su.mohammed@qu.edu.sa (M.S.M.S.); 5 Department of Biochemistry, Faculty of Medicine, Al-Azhar University, Assiut, 71524, Egypt; 6 Department of Plant Production and Protection, College of Agriculture and Veterinary Medicine, Qassim University, Qassim 51452, Saudi Arabia; ruessam2@yahoo.com; 7 College of Pharmacy, Qassim University, Qassim 51452, Saudi Arabia; alghulayqeh.osama@gmail.com; 8 Biochemistry Department, Faculty of Pharmacy, Al-Azhar University, Cairo 11884, Egypt; Kandil.yasser@azhar.edu.eg; 9 Pharmacological and Diagnostic Research Centre, Faculty of Pharmacy, Al-Ahliyya Amman University, Amman 19328, Jordan

**Keywords:** *Salsola cyclophylla*, halophyte, trace elements, LC-MS, anti-oxidant, anti-inflammatory, analgesic activity, cytotoxicity, toxicity evaluation

## Abstract

*Salsola cyclophylla*, an edible halophyte, is traditionally used for inflammation and pain. To confirm the claimed anti-inflammatory and analgesic properties, a detailed study on respective pharmacological actions was undertaken. The activities are contemplated to arise from its phytoconstituents. The LC-MS analysis of *S. cyclophylla* 95% aqueous-ethanolic extract revealed the presence of 52 compounds belonging to phenols, flavonoids, coumarins, and aliphatics class. A high concentration of Mn, Fe, and Zn was detected by atomic absorption spectroscopic analysis. The ethyl acetate extract showed the highest flavonoid contents (5.94 ± 0.04 mg/g, Quercetin Equivalents) and Fe2^+^-chelation (52%) potential with DPPH radicals-quenching IC_50_ at 1.35 ± 0.16 mg/mL, while the aqueous ethanolic extract exhibited maximum phenolics contents (136.08 ± 0.12 mg/g, gallic acid equivalents) with DPPH scavenging potential at IC_50_ 0.615 ± 0.06 mg/mL. Aqueous ethanolic extract and standard quercetin DPPH radicals scavenging’s were equal potent at 10 mg/mL concentrations. The aqueous ethanolic extract showed highest analgesic effect with pain reduction rates 89.86% (*p* = 0.03), 87.50% (*p* < 0.01), and 99.66% (*p* = 0.0004) after 60, 90, and 120 min, respectively. Additionally, aqueous ethanolic extract exhibited the highest anti-inflammation capacity at 41.07% (*p* < 0.0001), 34.51% (*p* < 0.0001), and 24.82% (*p* < 0.0001) after 2, 3, and 6 h of extract’s administration, respectively. The phytochemical constituents, significant anti-oxidant potential, remarkable analgesic, and anti-inflammatory bioactivities of extracts supported the traditionally claimed anti-inflammatory and analgesic plant activities.

## 1. Introduction

Genus *Salsola*, a part of the Chenopodiaceae plant family, also known as the Saltwort group of plants due to its high salt tolerance, can withstand salinity, dry conditions, and harsh desert climate. The genus is distributed globally in the arid and semi-arid zones, and a large number of plant species from the genus are edible, where some are also used as animal-feed [1]. The use of plants from the genus *Salsola* as an energy-provider, an immune booster, bone-density promoter, treating wasp-stings, smallpox, and Alzheimer’s disease are note-worthy [2]. Besides, several other pharmacological activities of the plants from this genus like antimicrobial [3], anti-oxidant [4], hepatoprotective [5], cytotoxic [6], and anti-cholinesterase [7] are reported. Due to the high nutritional value, these plants are consumed in many parts of the world [8].

The genus *Salsola* is represented by nine halophytic species in the Qassim region, Kingdom of Saudi Arabia [9], whereas *S. cyclophylla* is the most abundant species in the region. *S. cyclophylla* is a well-known plant to Bedouins and locals alike for its medicinal and other health benefits, including nutritional values [9]. The plant is also locally used as a camel-feed and is a good alternative food source in difficult and drought times [9,10]. For medicinal purposes, the plant is prepared as a tea and concoction for ameliorating various ailments, especially in managing inflammation and pain by the tribes and traditional healers alike. Locals also use the plant as a diuretic, laxative, and anthelmintic [11]. *S. cyclophylla* has been investigated for its volatile oil constituents and antimicrobial activity. The hexahydro-farnesyl acetone and benzoic acid esters were found as volatile oil’s major constituents. The oil also showed significant antimicrobial activity against *Staphylococcus aureus* and *Candida albicans* [10]. Apart from these, there are no other reports concerning the chemical and pharmacological investigations of *S. cyclophylla* growing in Saudi Arabia. However, several phytoconstituents are reported from other *Salsola* species, e.g., flavonoids, phenolic acids [12,13,14,15,16,17,18], sterols, hydroxy-fatty acids [19], alkaloids, organic acids [20], phenylpropanoids [21], and triterpenoidal saponins [22]. Among the bioactivities, the anti-oxidant and anti-microbial [12,13,19], anti-inflammatory [14], contraceptive [15], anti-nociceptive [17], and anti-diabetic [18] activities are reported for the plants extracts, fractions, and isolated compounds.

To complement our ongoing work on the locally-encountered halophytic plants, their constituents, and bioactivities in general, and the traditionally-claimed health-benefits and pharmacological properties, investigations on *Haloxylon, Cichorium, Tamarix, Ipomea*, *Zygophyllum*, *Styrax,* and *Suaeda* [10,23,24], together with *S. cyclophylla* were planned. The current study was designed to investigate the anti-inflammatory and analgesic activities of *S. cyclophylla* extracts and their related phytochemical constituents and anti-oxidant potentials. *S. cyclophylla* was of particular interest due to its immense nutritional and folklore-based anti-inflammation and pain removal applications.

## 2. Results and Discussion

### 2.1. LC-MS Analysis

Liquid chromatography-coupled-mass spectrometry (LC-MS) analysis was used to analyze the plant’s total extract obtained from 95%-aqueous ethanol obtained from the aerial parts of *S. cyclophylla*, and the identified compounds are listed in Table 1. A total of fifty-two constituents, representing 18.24% of the total LC-MS displayed constituents were identified, which included the tentatively identified constituents also. The molecular mass, mass spectral fragmentation patterns of the constituents, and standard samples were utilized for the purpose. Out of all the identified compounds, flavonoid aglycones and flavonoid glycosides, phenolics, and fatty acids were in a significant numbers at 53.84% (28 compounds), 23.08% (12 compounds), and 9.60% (5 compounds), respectively. The flavonoid and its glycosides and the phenolics constituted about 76.92% of the total identified products. Out of 52 compounds, seven constituents were identified through comparison with the standards injected during the LC-MS analysis. The triterpenes were 1.92% of these products, while diterpene, coumarin, alkaloid, disaccharide, other aromatic entities, and aliphatics-natured compounds, other than fatty acids, constituted, each, at 1.65% of the characterized constituents. These miscellaneous categories of di- and triterpenes, coumarin, alkaloid, disaccharide, dicarboxylic acid, aromatic entities, and aliphatics together constituted 11.56% of all the identified components from the 95% aqueous-ethanolic extract. Quantitatively, the flavonoids and phenolics were the major constituents. The high occurrence of the flavonoids and phenolic derivatives were also corroborated by the quantitative analysis of the dried extracts for categories of the compounds in the aqueous-ethanolic extract, which showed the major presence of flavonoids and phenolics in different extracts. From the LC-MS analysis, kaempferol-3-O-rutinoside (retention time 4.22 min), scutellarein-7-glucuronide (4.60 min), hesperidin (4.90), 8-prenyl naringenin (5.90 min), isobavachin (7.35 min), artocarpin (8.69 min), genistin (8.80 min), corymbosin (12.71 min), and pseudobaptigenin (13.68 min) were identified as the flavonoid and flavonoid glycosides derivatives. The palmitoleic acid (25.50 min), linoleic acid (27.91 min), oleic acid (28.23 min), and Octadecanoic acid (29.94 min) were parts of the constituents of the fatty acid. The medicagenic acid (castanogenin) was identified as the triterpenoidal component from the 95% aqueous-ethanolic extract analysis. The caffeic acid, a well-known anti-oxidant, and its derivative, caffeic acid phenethyl ester, were also present in the 95% aqueous-ethanolic extract and identified through their comparisons with the standard samples ran during the LC-MS chromatographic analysis. The 4-methyl umbelliferone was the sole product identified as the coumarin class of compound.

The plant 95% aqueous-ethanolic extract yielded flavonoids, phenolics, alkaloids, and sterols as also confirmed by the subsequent qualitative analysis, while the phenolics and flavonoids were quantified as gallic acid and quercetin equivalents for the *S. cyclophylla*. These contents are in higher proportions in the *S. cyclophylla*. The abundance of flavonoids and phenolics compounds contributed immensely to the plant’s anti-oxidant potential, and perhaps the ensuing better analgesic and anti-inflammatory bioactivities. The LC-MS analysis also endorsed the rich nature of the *S. cyclophylla* plant in terms of varying chemical classes of compounds present in it, whereby nearly two-third of all the identified products are flavonoids and phenolics in nature.

### 2.2. Trace Elements Analysis

As a halophytic plant, the *S. cyclophylla* is a potential source of essential minerals and a probable source of accumulated toxic heavy metals from the harsh and elements-rich sandy and saline environment [25]. Since the salinity enhances the mobility of heavy metals in this type of soil [26], the identification of *S. cyclophylla* trace elements was important from the viewpoint that the plant is used as an animal feed in its growing areas. It was pertinent to investigate trace elements’ presence because the trace elements could also play a role in the bioactivity, oxidative stress, and the anti-oxidant defensive mechanisms of the plant against environment-triggered oxidative damages [27]. As an animal feed, *S. cyclophylla* powder contains a substantial concentration of magnesium (Mg) at 2.90 mg/kg (Table 2), which is higher than *S. tomentosa* and less than *S. rigida*, and both the plants are found in different, but antagonistic environments as reported in a previous study [28].

The element magnesium presents in all tissues, especially the bones, and has an important role in activating many enzymes related to DNA replication. Additionally, elemental zinc stimulates insulin secretion and increases tissue sensitivity to insulin action, which could be used as an anti-diabetic agent [29]. It is noteworthy that toxic heavy metals, lead, and cadmium was also detected in *S. cyclophylla* at concentrations of 319 µg/kg and 109 µg/kg, respectively. In contrast, the maximum permissible limits of lead and cadmium in medicinal plants, determined by the World Health Organization (WHO) are 10 mg/kg and 0.3 mg/kg, respectively [30]. The current findings showed that the levels of heavy metals lead and cadmium in *S. cyclophylla*, growing locally are far below the prescribed toxic limits, and it is an indication of the plant’s safety.

According to the analysis, the trace elements, i.e., iron, copper, zinc, and manganese, were highly accumulated in the *S. cyclophylla* compared to other plants of the genus *Salsola*, e.g., *S. tomentosa* and *S. rigida* [28], and which were at their concentrations of 172.60, 7.81, 49.66, and 9.95 mg/kg, respectively (Table 2). These trace elements have essential roles in anti-oxidant enzymes’ functioning by affecting the body’s redox-related signaling pathways [31]. They also form the parts of nutritional standards for the presence of essential elements as the required minerals for the body, supplied by diets, to both humans and animals [32].

### 2.3. Phytochemical Screening of the Plant Constituents: Qualitative Analyses

Phytochemical screening of the *S. cyclophylla* extracts was conducted using qualitative chemical test methods. The results confirmed the presence of phenolics and flavonoids in the chloroform, ethyl acetate, and aqueous-ethanolic extracts. The major presence of flavonoids was detected in the chloroform and ethyl acetate extracts, which indicated that the flavonoids present in these extracts are structurally different from the flavonoids detected in the aqueous-ethanolic extract. The aqueous-ethanolic extract-based flavonoids, in all prospects, contained maximum numbers of free hydroxyls and glycosyl units as part of their structures. These constituents are in lower concentrations, while that is not the case with the chloroform and ethyl acetate extracts, where, considerably, the flavonoids, in most probability, are structurally devoid of the glycoside units, and thus structurally different. These non-glycosidic flavonoid entities are comparatively in higher concentrations in the plant and accumulated in the chloroform extract (Table 3). The alkaloidal constituents were detected in the plant’s ethyl acetate extract by Mayer’s and Dragendorff’s reagents-based test. Moreover, the qualitative testing results for the classes of compounds present in the plant also indicated the existence of sterols in the chloroform extract, while aliphatics and the waxy materials were accumulated in the *n*-hexane extract.

### 2.4. Total Phenolics and Flavonoids Contents Analyses

The phenolics and flavonoids have imperative health benefits with nutritional values and play an important role in the halophytes’ protection against oxidative stress resulting from the saline and other harsh-conditioned environments of the plant habitat [33]. Therefore, the qualitative and quantitative determinations of the presence and concentrations of phenolics and flavonoids in halophytes have previously been extensively conducted [34].

The current study also quantitatively estimated the phenolics and flavonoids contents present in the *S. cyclophylla*. Most phenolic compounds were detected in the aqueous-ethanol extract (136.08 ± 0.12 mg/g, GAE, gallic acid equivalent), indicating the high polarity of these compounds. Perhaps the products being multi-hydroxylated and glycosylated in their structural specifications were extracted together with other high-polar molecular entities that are non-reactive to the assay reagent and accounted for their presence in the extract. The phenolics and flavonoids are at 136.08 ± 0.12 mg/g and 3.19 ± 0.03 mg/g of concentrations, respectively of the dry extract per gram of the aqueous-ethanolic extract. The flavonoids quantitatively measured in higher concentration in the ethyl acetate extract (5.94 ± 0.04 mg/g) and chloroform extract (5.38 ± 0.00 mg/g), followed aqueous-ethanolic extract (3.19 ± 0.03 mg/g), and by the lesser concentrations in the *n*-hexane extract (1.68 ± 0.01 mg/g), all as QE (quercetin equivalents). The phenolics were detected in the *n*-hexane (32.70 ± 0.01 mg/g), chloroform (85.38 ± 0.04 mg/g), ethyl acetate (32.40 ± 0.02 mg/g), and aqueous-ethanolic (136.08 ± 0.12 mg/g) extracts, all as gallic acid equivalents (GAE). Most of the phenolic compounds were detected in the aqueous-ethanolic extract (136.08 ± 0.12 mg/g GAE), indicating the high polarity of these compounds and suggesting that they are structurally multi-hydroxylated and also glycosylated in nature. The phenolic contents were in comparatively lower concentrations in the *n*-hexane, chloroform, and ethyl acetate extracts, as compared with the aqueous-ethanolic extract, all being at 32.70 ± 0.01, 85.38 ± 0.04, 32.40 ± 0.02, and 136.08 ± 0.12 mg/g concentrations as GAE, respectively. The flavonoids found in the aqueous-ethanolic extract of *S. cyclophylla* (3.19 ± 0.03 mg/g QE) are of strong hydrophilic nature. The comparatively higher levels of the flavonoids in the ethyl acetate (5.94 ± 0.04 mg/g QE), and chloroform (5.38 ± 0.00 mg/g QE) extract, indicated the moderate polarity of these flavonoids, and possibly the presence of fewer glycosylated flavonoid structural entities in these extracts than the aqueous-ethanolic extract.

### 2.5. DPPH Scavenging and Iron-Chelating Activities of S. cyclophylla Extracts

The halophytic plants are exposed to produce anti-oxidant enzymes and secondary metabolites to overcome the oxidative damage of their harsh environment of sandy and saline conditions. Furthermore, the plants’ anti-oxidant capacity and their products play major roles in their anti-inflammatory and analgesic activities [34,35]. *S. cyclophylla* extracts were evaluated for their anti-oxidant activity by two different assay methods. The scavenging activity of the extracts for free radicals was examined against DPPH stable radicals. As the ions potentiate the oxidative degenerations in the body [36], the necessity to check the *S. cyclophylla* extracts in chelating the ferrous ions was undertaken and determined by the ferrozine-based assay.

**Figure 1:**
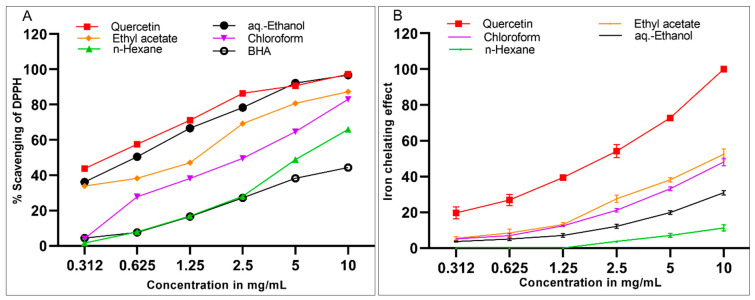
Anti-oxidant potentials of *S. cyclophylla* extracts are expressed as scavenging reactivity towards DPPH free radicals (**A**) and the iron-chelating effects (**B**). Statistical significance was performed by two-way ANOVA (*p* < 0.0001). According to Tukey’s method, the extracts groups at all the measured concentrations were significantly different (*p* < 0.0001) compared to quercetin in both Figures (**A**,**B**).

The obtained results (Figure 1) showed that the significant anti-oxidant activity of *S. cyclophylla* was compatible with the plant’s halophytic nature, and this fact of higher anti-oxidant activity has also been encountered in other halophytes [35,37]. These results revealed that the aqueous-ethanolic extract (IC_50_ 0.615 ± 0.06 mg/mL) was the best extract in scavenging the DPPH-free radicals. The DPPH radicals scavenging potentials of the aqueous-ethanolic extract and the standard quercetin were approximately 37% and 44%, respectively, at the lowest concentration (0.0312 mg/mL). The ethyl acetate (IC_50_ 1.354 ± 0.16 mg/mL), chloroform (IC_50_ 2.395 ± 0.24 mg/mL), and *n*-hexane (IC_50_ 4.958 ± 0.50 mg/mL) extract showed concentration-dependent lesser DPPH-radicals scavenging effects as compared to the aqueous-ethanol extract. This result concurs well with our assessment of the phenolics and flavonoid contents of the extracts, as higher levels of phenolics were found in the aqueous-ethanolic extract, and also together, the combined concentrations of the phenolics and flavonoid contents were at the highest levels in the aqueous-ethanolic extract wherein the constituents possessed strong potentials for DPPH-radicals scavenging activity. Figure 1A also exhibited that all the *S. cyclophylla* extracts were significantly more effective as DPPH free radical scavengers than the BHA standard, except the *n*-hexane extract, which was, comparatively, similar to the BHA and lower than other extracts in the anti-oxidant potentials.

The iron-chelating activity of the plant extracts containing flavonoids, phenolics, and alkaloids is well established [38]. The presence of these contents in the *S. cyclophylla* was the most important factor responsible for the ethyl acetate extract’s higher iron-chelating activity, followed by the chloroform extract. The higher chelating activity of the ethyl acetate extract was attributed to the presence of alkaloids and flavonoids in the extract. The alkaloidal constituents have specific sites for iron-chelation as compared to the chloroform extract products, which were devoid of the presence of any significant concentration of the alkaloids. The iron-chelating activity (Figure 1B, Table 3) of quercetin was highest at 10 mg/mL, followed by the 52% chelation capacity of the ethyl acetate extract, which contained higher concentrations of the flavonoids and alkaloids. The minimum concentration at which the iron-chelating activity was exhibited for all the extracts was slightly over 1.25 mg/mL of the concentrations (Figure 1B). Moreover, the pure quercetin demonstrated dose-dependent and significantly higher (*p* < 0.0001) iron-chelating activity as compared to all the *S. cyclophylla* extracts. Wherein the ethyl acetate and chloroform extracts, respectively, were more reactive at all the tested concentrations than the other two extracts, i.e., aqueous-ethanol and *n*-hexane extracts, and that presumably was supposed to be due to the comparatively higher contents of non-glycosylated flavonoids in the ethyl acetate and chloroform extracts (5.94 and 5.38 mg/g QE, respectively).

Pearson’s analysis demonstrated a positive correlation between the total flavonoid contents in different extracts and their iron-chelating activities (r = 0.98, *p* < 0.01). However, no correlation has been observed between the flavonoid contents and the DPPH scavenging effect of the extracts. Additionally, no significant correlations were demonstrated among the phenolics contents of the extracts with DPPH scavenging and iron-chelating effects.

The flavonoids and phenolics phytoconstituents possess both anti-oxidant and anti-inflammatory activities. The anti-oxidant activity of *S. cyclophylla* can also be correlated to its significant presence of trace elements, including Fe, Cu, Zn, and Mn [39]. Interestingly, a recent study reported the increased gene expression and serum activities of anti-oxidant enzymes after the ingestion of selenium/zinc probiotics in rats [40].

### 2.6. Acute Toxicity Study

The animals, rats, were administered, separately, a single dose of 5000 mg/kg of *n*-hexane, chloroform, ethyl acetate, and aqueous-ethanolic extracts of *S. cyclophylla* to assess acute toxicity (*n* = 5/group). For the initial 3 days, the animals were monitored daily for any visible signs of toxicity. The acute toxicity, including mortality, was recorded for 2 weeks. None of the extracts administered groups displayed any visible signs of toxicity in the first 3 days, while one animal each died on days 8 and 10 for n-hexane, and ethyl acetate extracts groups, respectively. According to the OECD guidelines, the dose is considered toxic if more than one animal dies in any group. According to the Hedge and Sterner scale, the given dose was determined to be practically non-toxic, and one-tenth of the dose was selected for the analgesic and anti-inflammatory bioactivities evaluations [41,42].

### 2.7. Analgesic Activity

The analgesic activities of the extracts were assessed using the hot plate, a centrally mediated pain-induced mice model. The central and peripheral analgesic effects were assessed using a hot-plate and acetic-acid writhing methods, respectively. The hot-plate technique evaluated the time taken by the animal to feel the sensation of the pain generated by the response through initiation at the peripheral nerve endings, and the propagation of the pain sensation to the brain. The animals that received the aqueous-ethanolic extract showed comparable analgesia to the diclofenac group at all time points compared with the olive-oil group control. For the aqueous-ethanolic extract group, the inhibition of pain increased by 89.86% (*p* = 0.03), 87.50% (*p* < 0.01), and 99.66% (*p* = 0.0004) after 60, 90, and 120 min, respectively, compared with the olive-oil control group (Table 4).

**Figure 2:**
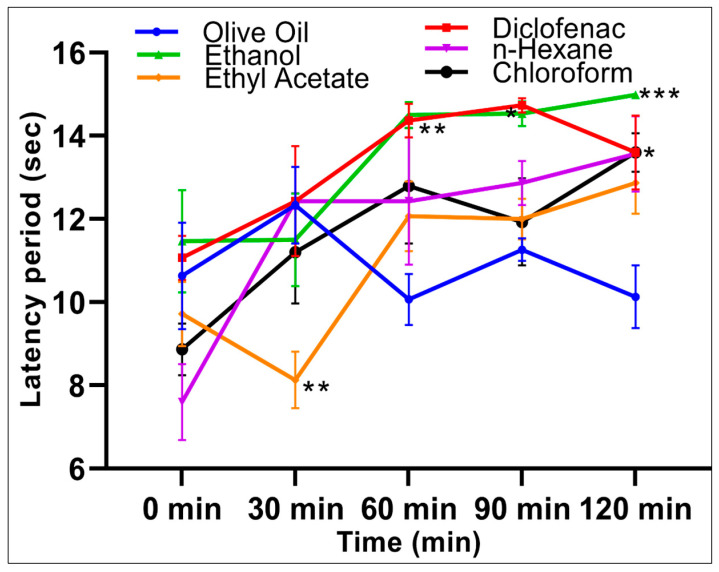
Analgesic effects of the *S. cyclophylla* extracts as assessed by the hot-plate pain-induction method in mice. Values are denoted as mean ± SEM, *n* = 6 animals/group. The statistical significance was performed via a one-way ANOVA followed by a posthoc test using Tukey’s multi-group comparison: * *p* < 0.05, ** *p* < 0.01, and *** *p* < 0.001 compared to the olive oil control group. For example, at 120 min compared to the olive oil group, the diclofenac, n-hexane, and chloroform extracts groups were significantly different * *p* < 0.05.

The *n*-hexane and chloroform extracts showed significant (*p* < 0.05) pain inhibition potentials, with a reduction of 70.55% and 71.23%, respectively, at 120 min as compared with the negative control group, while the ethyl acetate extract reduced the pain by 56.16% after 120 min (Figure 2). The extracts’ analgesic effects decreased at 30 min by −31.25%, −157.50%, and −42.50% for aqueous-ethanolic, ethyl acetate, and chloroform extracts, respectively, compared to the control. The concurrent slow onset of the actions of the extracts indicated that the pain threshold did not reach its limit through the depression of the pain receptors in the brain, or there is a delay in the firing of the nociceptors, or the early and late nociceptive phases, which showed the dissimilar pain effects [43,44]. The aqueous-ethanolic extract group showed significantly increased pain inhibitions at 60, 90, and 120 min. In comparison, the ethyl acetate and chloroform extracts showed biphasic responses with the peak analgesic activity at 60 min, followed by dipping in the analgesic activity at 90 min. The highest analgesic activity of 56.16% and 71.23%, respectively, were observed at 120 min.

However, upon considering the pain pathways, concluded from the results obtained from the hot-plate method, the analgesic activity of *S. cyclophylla* can be attributed to the inhibitions of inflammatory mediators and depression of the pain-inducing receptors [45]. The analgesic effect may also be observed due to the comparatively higher contents of Mg, Cu, and Zn in the plant [46], as a previous study has detailed the molecular mechanism of the pain-relieving effects of dietary zinc supplementation in mice [47].

### 2.8. Anti-Inflammatory Activity

The anti-oxidant activity of other plant extracts and the effects on inflammation has been previously studied in detail. The oxidative damage has been observed to be related to inflammation [45,48]. It is also known that increased ROS generation at the inflammation site causes oxidative stress and perhaps consequent tissue injury. The ROS-induced inflammation is detrimental, and if stress is unchecked, it may lead to several pathologies [49,50]. The phenolics, flavonoids, and other potent anti-oxidants have conclusive roles to play [45,51,52]. The anti-oxidants treatment-based improvements in inflammatory conditions and the report of the plant extracts’ effectiveness in ameliorating the inflammatory conditions are known [53,54].

During the current study, the carrageenan-induced paw edema method was used to assess the anti-inflammatory activity of the *S. cyclophylla* extracts in comparison with a negative vehicle (olive oil) control and a positive (diclofenac) control. The carrageenan-induced inflammation control, with lowest paw-volume maintenance at 1, 2, 3, and 6 h, and at par with the diclofenac group after 24 h by the aqueous-ethanolic extract substantiated the roles of the anti-oxidant constituents in the extract, i.e., phenolics and flavonoids, which together are at the highest concentrations (136.08 ± 0.12 mg/g, 3.19 ± 0.03 mg/g, GAE, and QE, respectively) in the extract. However, for the experimental animals receiving the negative vehicle, edema volume increased after carrageenan induction at 0 h (1.03 ± 0.01), and reached the peak after 6 h (1.54 ± 0.02), followed by a gradual decrease over the 24 h period to 1.47 ± 0.01 of the paw volume. For the aqueous-ethanolic extract group, the inhibition of inflammation increased by 16.79% (*p* < 0.001) at 1 h compared with the negative control, and showed a peak inhibition of inflammation at 41.07% (*p* < 0.0001) after a 2 h period (Table 5). The inhibitions of inflammation gradually decreased to 24.82% (*p* < 0.0001) and 9.39% (*p* < 0.05) after 6 and 24 h, respectively, as compared with the olive-oil control group. These results were similar to the diclofenac control group and concurred with our findings that the aqueous-ethanolic extract was richest in the anti-oxidants phenolics and flavonoids, and the phytoconstituents played its role well.

**Figure 3:**
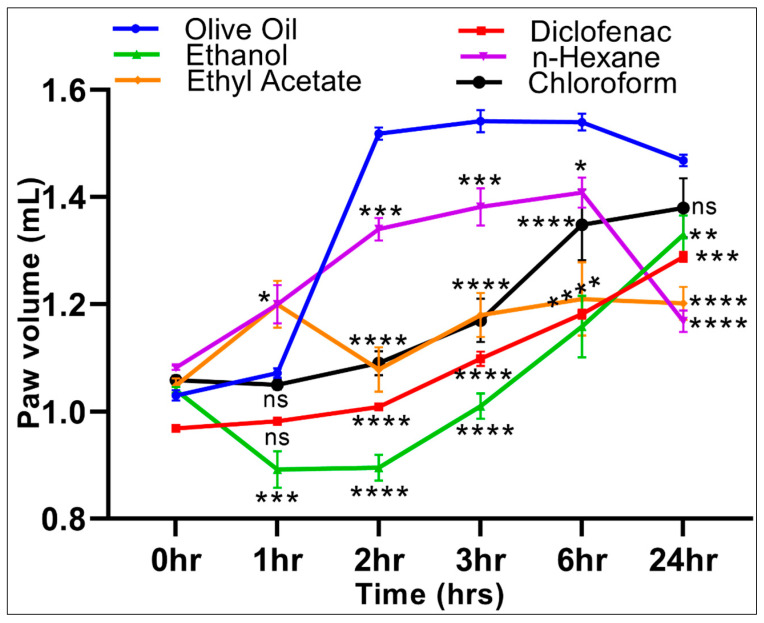
Anti-inflammatory activities of *S. cyclophylla* extracts assessed via carrageenan-induced paw edema in rats. The values denoted are mean ± SEM, *n* = 6 animals/group. The statistical significance were performed via a one-way ANOVA followed by a posthoc test using Tukey’s multi-group comparison: ns: not significant, * *p* < 0.05, ** *p* < 0.01, *** *p* < 0.001, and **** *p* < 0.0001 compared to the olive oil group. For example, at 2 h compared to the olive oil control group, the diclofenac, aqueous-ethanolic, ethyl acetate, and chloroform groups were significantly different, * *p* < 0.0001.

Conversely, the n-hexane extract group showed a significant increase in inflammation, 11.92% (*p* < 0.05) at 1 h, compared with the negative control group observations, followed by an inhibition of inflammation of 11.71% (*p* < 0.001), 10.37% (*p* < 0.05), and 20.44% (*p* < 0.0001) at 2, 3, and 24 h, respectively, as compared with the olive-oil group. The chloroform extract suppressed edema by 28.18% (*p* < 0.0001) and 24.14% (*p* < 0.0001) after 2 and 3 h, respectively, compared with the negative control. Meanwhile, the ethyl acetate extract group showed a significant increase of inflammation of 11.92% (*p* < 0.05) at 1 h compared with the olive-oil control group, followed by the inhibition of inflammation at 28.99% (*p* < 0.0001), 23.49% (*p* < 0.0001), 21.43% (*p* < 0.001), and 18.09% (*p* < 0.0001) at 2, 3, 6, and 24 h, respectively, compared with the olive-oil group (Figure 3).

The reactive oxygen species (ROS), free radicals, and other unstable molecular entities cause damage to the cells. The inflammation entails an increase in ROS, which is countered by radical scavengers, mainly the plants’ flavonoids and phenolics phytoconstituents. In the current study, the extract containing higher concentrations of phenolics and flavonoid compounds worked as a better radical scavenger, which was well correlated with the anti-inflammatory and analgesic effects of the extracts as reported earlier [45]. The higher concentrations of phenolics and flavonoids contents in the *S. cyclophylla* extracts indicated the strong anti-inflammatory potency, which has been reported to be mediated through blocking the prostaglandins (PG) and cyclooxygenase (COX) receptors [55]. The flavonoids are known to inhibit the nuclear factor’s transcription by inhibiting the kinases present in the signal transduction pathway [56]. The analgesic effects of the halophytic plant, *S. cyclophylla*, may also be better exhibited due to the comparatively higher presence of Mg, Cu, and Zn contents in the plant, as compared to other normal-environment habited plants. Additionally, the molecular mechanism of pain-relieving effects after dietary zinc supplementation in mice is known [47].

The current study involved investigating the inhibition of inflammation using carrageenan-induced inflammation, a preliminary screening inflammatory model producing acute and reproducible inflammation. The induced inflammation occurs in two stages. The primary stage (1–2 h) involves the production of chemical mediators, i.e., histamine, serotonin, and bradykinin, and the second stage (3–6 h) involves an increase in PG, leukotrienes, and free radicals, leading to significant vascular permeability, together with an influx of neutrophils that lead to edema formation through a build-up of plasma fluid. The flavonoids are known inhibitors of inflammation in both stages through cytokines, and COX [43,57].

The aqueous-ethanolic and ethyl acetate extracts showed a significant reduction of inflammation in both stages, while the n-hexane and chloroform extracts showed significant anti-inflammatory activity during stage one only, and that too was dependent upon the responses time. The effects of the aqueous-ethanolic extract were comparable to the diclofenac (sodium), which is known to exhibit higher binding affinity to the accumulated proteins in the inflamed tissues at significantly higher levels, as compared to the plasma, which is also known to act by inhibition of PGs through blockade of COX [58]. The plant’s anti-inflammatory effect is, presumably, added up due to its zinc content [40].

### 2.9. Plausible Inflammation Biomechanistics and Roles of S. cyclophylla

The ROS, free radicals and unstable molecules cause damage to cells. The inflammation may increase in ROS, which is needed to be countered by the radical scavengers. In the current study, the extract containing higher concentrations of phenolics and flavonoid compounds together (aqueous-ethanolic) worked as a better radical scavenger, which correlated well with the corresponding extract’s anti-inflammatory effects. The high concentrations of phenolics and flavonoid compounds in the *S. cyclophylla* extract indicated their strong anti-inflammatory potential, which likely is mediated through inhibitions of prostaglandin (PG) and cyclooxygenase (COX) receptors. The flavonoids are known to inhibit the nuclear factors transcription, cytokines, and COX, and help to inhibit chemical mediators’ production from stopping the edema formation [59,60]. The effect of the aqueous-ethanolic extract was found to be comparable to the diclofenac, a known ligand with a higher binding affinity to the proteins in the inflamed tissues and known to work by the inhibitions of PGs [61].

### 2.10. Cytotoxicity Evaluation of the 95% Aqueous-Ethanolic Extract

**Figure 4:**
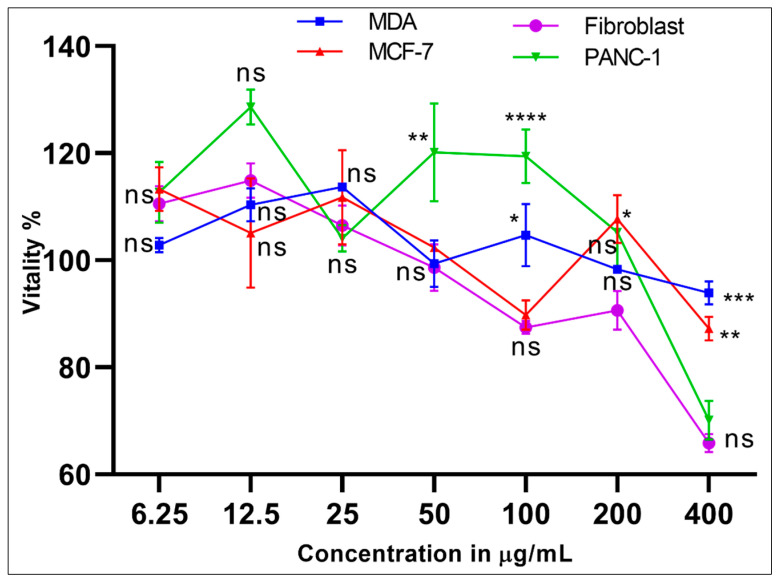
The cell vitality of the *S. cyclophylla* 95% aqueous-ethanolic extract. Values denoted are mean ± SEM. The statistical significance was performed via a one-way ANOVA followed by a posthoc test using Tukey’s multi-group comparison: ns: not significant, * *p* < 0.05, ** *p* < 0.01, *** *p* < 0.001, and **** *p* < 0.0001 compared to fibroblasts. For example, at 50 µg/mL compared to the fibroblasts, the MDA and MCF-7 are not significant, while PANC-1 is significant (** *p* < 0.01).

The MDA (M14 melanoma derived epithelial breast cancer cells), MCF-7 (Michigan Cancer Foundation-7, breast cancer cells), PANC-1 (human pancreatic cancer cell line isolated from a pancreatic carcinoma of ductal cell origin), and the normal human fibroblast cell lines were cultured and used in the *S. cyclophylla* 95% aqueous-ethanolic extract’s cytotoxicity evaluations to check the anti-cancer properties and the cell toxicity. The concentrations at 400 μg/mL were diluted serially to 6.25 μg/mL, and the cell proliferation was tested. The cells’ vitality was compared with the normal fibroblast cells with doxorubicin as the positive control, and the cell vitality levels for the higher concentration, from 400 to 50 μg/mL, showed moderate to low effects. At concentrations below 50 μg/mL, as observed at 25 μg/mL and 12.5 and 6.25 μg/mL, the cell vitality differences were insignificant for cytotoxicity purposes (Figure 4).

The work reports the verification of the traditionally claimed strong analgesic and anti-inflammatory biological properties of the halophytic plant, *S. cyclophylla*, which is also used as salad, emergency food, and animal-feed regularly in the central areas of the Saudi Arabian Desert. The LC-MS analysis confirmed the presence of major phytoconstituents of the phenolics, and flavonoids’ nature. The presence of comparatively higher concentrations of trace elements, as compared to non-halophytic plants, was also recorded. The anti-oxidant, anti-inflammatory, and analgesic activities were investigated, and the presence of higher phenolics and flavonoid contents, and the trace elements seemed to be playing a part in the claimed strong pharmacological efficacy of the plant. The aq.-ethanol extract, rich in phenolics and flavonoids, showed comparatively better anti-oxidant, analgesic, and anti-inflammatory properties, followed by the chloroform and ethyl acetate extracts, also forming the rich anti-oxidant base of the plant. The role of phytoconstituents, the potential of quenching the reactive oxygen species through strong anti-oxidant character, and the highly concentrated presence of essential trace elements form the basis of the plant’s claimed health benefits. No significant anti-cancer activity or cytotoxicity was found, and the plant’s safety profile evaluation confirmed the safety of the plant used by the traditional medicinal practitioners. Based on the findings, the plant was found to be safe for human and animal consumptions validated under conditions of the tested dose, its frequency, and period of use.

## 3. Materials and Methods

### 3.1. Plant Materials Collection and Extraction Procedure

The plants were collected from Al-Fuwayliq City in the Qassim region (GPS: 26°33′37.9″ N 42°48′27.1″ E) during October 2018 and were identified as *S. cyclophylla* Baker by the institutional taxonomist. A voucher specimen was stored in the Pharmacognosy Laboratory, College of Pharmacy, Qassim University (#79). The plant’s aerial parts were dried in the shade at room temperature (RT) and processed to a coarse powder by a mechanical grinder. The powdered material (1 kg) was extracted (1500 mL × 3) in n-hexane (22.48 g), chloroform (87.60 g), ethyl acetate (112.05 g), and aqueous-ethanol (3:7, water: ethanol) (117.29 g) in sequence at RT by the maceration method. Additionally, 100 g of the plant powder was directly extracted by 95%-aqueous-ethanol to obtain the 95% aqueous-ethanolic extract used for the LC-MS and cytotoxic evaluations of the plant material. The 95% aqueous-ethanolic extract and various other extracts obtained by the sequential extractions of the plant powder were filtrated and dried under vacuum at <40 °C until complete dryness. The dried extracts were stored at −20 °C for further use.

### 3.2. Qualitative Analysis of the Extracts

The flavonoids, sterols, and alkaloids were detected using reported qualitative analysis methods [62].

### 3.3. Trace Elements Analysis

The trace elements analysis was performed on the dried plant powder according to the method described by Jones, Jr. [63]. Fresh plant material, aerial parts, was dried at 70 °C for 48 h and sieved to <0.15 mm (millimeter) and <0.5 mm using a stainless-steel mill. The sifted material (0.5 g) was digested in a mixture containing concentrated HNO_3_, HCIO_4_, and H_2_SO_4_ (7:2:l). The Fe, Mn, Zn, Mg, Cu, Cd, and Pd contents were measured by inductively coupled plasma atomic emission spectroscopy (ICP-OES; Model iCAP 7400 Duo, serial IC 74DC144208; Thermo Fisher Scientific, Shanghai, China). The concentrations of the trace elements in the plant powder were calculated from the calibration curves prepared for each element and calculated as µg per kg of the plant powder.

### 3.4. LC-MS Analysis

All the reagents and solvents, i.e., acetonitrile, methanol, water, and formic acid were HPLC grades. Stock solutions of standard compounds were prepared by dissolving the appropriate amount of materials in analytical grade dimethyl sulfoxide (DMSO), diluted with acetonitrile, and identifying *m*/*z* (mass/charge), molecular mass (atomic mass unit), and retention times (in minutes) of all the phytoconstituents. The dried-gel-like 95% aqueous-ethanolic extract (1 mg) was dissolved in 2.0 mL DMSO, then completed to 50 mL by acetonitrile addition, followed by centrifuging at 4000 rpm for 2.0 min, 1.0 mL was taken and transferred to an autosampler, and 3.0 µL was injected.

#### 3.4.1. LC-MS Instrumentation and MS Parameters

Bruker Daltonics (Bremen, Germany) Impact II ESI-Q-TOF System equipped with Bruker Daltonics Elute UPLC system (Bremen, Germany) was used for screenings of the compounds. The standards for identifying the *m*/*z* with high-resolution Bruker TOF MS and the exact retention times of each analyte were used as obtained during the chromatographic analysis. This instrument was operated using the Ion Source Apollo II ion funnel electrospray source. The capillary voltage was 2500 V, the nebulizer gas was 2.0 bar, and the dry gas (nitrogen) flow rate was 8 L/min with the dry temperature at 200 °C. The mass accuracy was <1 ppm; the mass resolution was 50,000 FSR (full sensitivity resolution), and the TOF repetition rate was up to 20 kHz.

#### 3.4.2. LC-MS: Sample Preparations, Experimental Conditions, and Analysis

The unknown sample was dissolved in 2.0 mL of DMSO, then completed to 50 mL by acetonitrile addition, and centrifuged at 4000 rpm for 2.0 min, 1.0 mL from it was taken and transferred to an autosampler where 3.0 µL was injected. Standards were used for the identification of *m*/*z* and retention times of all the contents. The chromatographic separation was performed on 120, C18 reverse-phase column, 100 × 2.1 mm size, 1.8 µm (120 A°) from Bruker Daltonics (Bremen, Germany) at 30 °C, and autosampler temperature of 8.0 °C with a total run time of 35.0 min using a gradient solvent system. For the positive mode, chromatographic analyses were performed using gradient elution with eluent B composed of methanol with 5 mM ammonium formate and 0.1% formic acid, and eluent A composed of water-methanol (90:10) with 5 mM ammonium formate and 0.1% formic acid. The compounds separations were performed using a gradient elution of B from 0.0 to 0.1 min with 4.0% of B, from 0.1 to 1.0 min with 18.3% of B, from 1.0 to 2.5 min with 50% of B, from 2.5 to 14.0 min with 99.9% of B, from 14.0 to 16 min with 99.9%, and then returning to 4% of B from 16.1 to 20.0 min, 18.3% from 20.1 till last minute. For the negative mode, the chromatographic analyses were performed using gradient elution with eluent B composed of methanol with 5 mM ammonium acetate, and eluent A composed of water/methanol (99:1) with 5 mM ammonium acetate. The compounds separations were performed using a gradient elution of B from 0.0 to 0.1 min with 4.0% of B, from 0.1 to 1.0 min with 18.3% of B, from 1.0 to 2.5 min with 50% of B, from 2.5 to 14.0 min with 99.9% of B, from 14.0 to 16 min with 99.9% of B, and then returning to 4% of B from 16.1 to 20.0 min, and 18.3% from 20.1 till last of the run.

### 3.5. Total Phenolics Contents Analysis

The total phenolics contents of the *S. cyclophylla* extracts were assayed as the gallic acid equivalent (GAE) using the Folin–Ciocalteu reagent [64]. Briefly, 2.5 mL of diluted Folin–Ciocalteu reagent (1: 10, *v*/*v*, distilled water) was added to 1 mL plant extracts at a concentration of 1 mg/mL in methanol followed by the addition of 2 mL of saturated aqueous sodium carbonate solution. The mixture was vortexed and left at 40 °C in a water bath for 30 min before the UV–Visible (UV–Vis) absorbance was measured at 765 nm using a spectrophotometer (Model V-630; JASCO Co., Tokyo, Japan). The extracts’ total phenolics contents were expressed as mg/g of the dried extract using the gallic acid calibration curve. The results were obtained as an average of three independent measurements.

### 3.6. Total Flavonoids Contents Analysis

The extracts’ flavonoid contents were expressed as the total quercetin equivalent (QE) in mg/g for the dried extract and the experiments were performed according to the literature method [64]. In brief, 1 mL of plant’s (any) extract at a concentration of 1 mg/mL in methanol was mixed with 1 mL aluminum chloride (AlCl_3_, 2%, ethanol). The mixture was vortexed and allowed to stand for 1 h at RT, and the UV–Vis absorbance was measured at 440 nm using a UV–Vis spectrophotometer (Model V-630; JASCO Co., Tokyo, Japan). The extracts’ flavonoid contents were calculated from quercetin’s calibration curve as mg/g of QE equivalent. The results were obtained as an average of three independent measurements.

### 3.7. Anti-oxidant Potentials: Radical Scavenging Activities of the Extracts

The radicals scavenging activity of each extract was measured calorimetrically using DPPH (2,2-diphenyl-1-picrylhydrazyl) according to the described method [65] with little modifications. Briefly, 1.9 mL of serially diluted plant extract, standard quercetin, and butylated hydroxyl anethol (BHA) in ethanol were mixed separately with 0.1 mL DPPH solution (300 µM in ethanol). The mixtures were incubated in the dark at 30 °C for 30 min. The reductions in DPPH color due to the extracts/standard compounds compared to the standard solution of the DPPH alone were measured spectrophotometrically at 517 nm. The scavenging effects of each extract were calculated using the following equation, Equation (1):

(1) $$\mathrm{Scavenging}\%=\left(1-\frac{S_{\mathrm{ab}}}{B_{\mathrm{ab}}}\right)\times 100\;\;\;\;$$

*S*_ab_ and *B*_ab_ refer to the sample, and the blank absorbance, respectively.

### 3.8. Ferrous Ions (Fe^2+^) Chelating Activity of the Extracts

The abilities of the *S. cyclophylla* extracts to chelate ferrous (Fe^2+^) ions were determined by the method described in the literature [66] with certain modifications. *S. cyclophylla* extracts (1 mL, 10–0.312 mg/mL) in methanol were vortexed and incubated for 10 min with 100 µL of ferrous chloride (1 mM) before adding 200 µL of 5 nM ferrozine. The mixtures were incubated for 5 min, and reduction in the violet color of the Fe^2+^-complex with ferrozine compared to a blank were determined spectrophotometrically at 562 nm by a UV–Vis spectrophotometer. The chelating activity of each extract was calculated from the following equation, Equation (2):

(2) $$\mathrm{Chelating}\;\mathrm{activity}\%=\left[\frac{A_{b}-A_{s}}{A_{s}}\right]\times 100,$$

where *A*_b_ is the absorbance of the blank, and *A*_s_ is the absorbance of the sample. The mean results were obtained from triplicated independent experiments.

### 3.9. Pharmacological Studies

#### 3.9.1. Drugs and Chemicals

Diclofenac sodium tablets were purchased from a local pharmacy. Organic olive oil was purchased from Al-Jouf market, Kingdom of Saudi Arabia, and commercial-grade type 1 carrageenan was obtained from Sigma Aldrich, MO, USA.

#### 3.9.2. Acute Toxicity and Sample Size

The acute toxicity study was conducted according to the Organization for Economic Cooperation and Development (OECD) procedure for acute toxicity testing [42]. A dose limit of 5000 mg/kg for all the *S. cyclophylla* extracts (n-hexane, chloroform, ethyl acetate, and aqueous-ethanolic) was used on 10-week-old male Sprague Dawley rats (*n* = 5 per extract). The overnight fasted animals were weighed, and a single oral dose of 5000 mg/kg was administered. The animals were observed for abnormality in behavior and movement for the first 3 days and were under mortality watch for over 2 weeks period [42]. The required sample size was determined using mean ± SEM carrageenan-induced paw edema values between the untreated control group, and the reference treated group, based on a previously published report [20]. The calculated effect size d was 4.30 using a two-tail option on G Power V.3.1.9.4 software, Heinrich Heine University, Düsseldorf, Germany. To achieve a statistical power (1-β err prob) of 80%, and a specific α error probability of 0.05, the minimum required sample size in each group was *n* = 5 rats.

#### 3.9.3. Evaluation of Pharmacological Activities of *S. cyclophylla* Extracts

The study was performed in accordance with the Animal Research: Reporting: In Vivo Experiments (ARRIVE) statement [67]. Thirty-six Sprague Dawley rats (male, weighing 150–200 g, ten-weeks-old) for anti-inflammatory activity, and thirty-six Swiss albino mice (male, weighing 20–25 g, ten-weeks-old) for analgesic activity, were maintained in the animal facility at the College of Pharmacy, Qassim University, Kingdom of Saudi Arabia. The animals were acclimatized for 3–5 days before dosing with a 12 h light-dark cycle at 24 ± 2 °C and 67% humidity. Animals were fed on a standard chow diet obtained from First Milling Company in Qassim, Buraydah, Kingdom of Saudi Arabia, and water ad libitum. All the experimental protocols utilizing animals were approved (Approval ID 2019-CP-8) and conducted according to the ethical guidelines of the College of Pharmacy Animal Ethics Committee, Qassim University, Kingdom of Saudi Arabia.

#### 3.9.4. Evaluation of the Anti-Inflammatory Activities of *S. cyclophylla* Extracts

A total of thirty-six rats were divided randomly into six groups (*n* = 6 per group). The vehicle control received olive oil (20 mL/kg per os (p.o.)), and the positive control group of animals received diclofenac sodium (10 mg/kg p.o.). Groups 3–6 received an extract of *S. cyclophylla* (*n*-hexane, chloroform, ethyl acetate, or aqueous-ethanolic extracts, respectively; 500 mg/kg/day p.o.) for 1 week. On the last day of dosing, the animals were subjected to a carrageenan-induced rat paw edema assessment described by the winter group [68] to examine the plant’s anti-inflammatory potential extracts. Fifteen minutes after administering the doses to all the groups, edema was induced on the rat’s left hind paw by a sub-plantar injection of 0.1 mL carrageenan (1%, *w*/*v*) and the time was considered as zero hour. Inflammation in the carrageenan-injected foot at a specific mark around the ankle was measured using a plethysmometer at 0, 1, 2, 3, 6, and 24 h. The inhibitions of paw inflammations were expressed as paw volume in mL, and percentage inhibition was calculated for the time points 1, 2, 3, 6, and 24 h using the following equation, Equation (3):

(3) $$\mathrm{Inhibition}\;\mathrm{of}\;\mathrm{edema}\%=\left(\frac{T-T_{0}}{T\;}\right)\times 100,$$

where *T* is the control group paw volume and *T*_0_ is the paw volume for the treatment groups.

#### 3.9.5. Evaluation of the Analgesic Activities of *S. cyclophylla* Extracts

A total of thirty-six mice were randomly divided into six groups (*n* = 6 per group). The first group of animals served as vehicle control, and received olive oil (20 mL/kg p.o.), while the second group of animals received diclofenac sodium (10 mg/kg p.o.) as a positive control. The groups 3–6 received an extract of *S. cyclophylla* (*n*-hexane, chloroform, ethyl acetate, or aqueous-ethanolic, respectively; 500 mg/kg/day p.o.) for 1 week. The analgesic activity of each extract was investigated on the last dosing day using the hot-plate method described by Eddy and Leimbach [69] with minor modifications. Each animal was placed individually on the hot plate to observe their response. The analgesic activity evaluation was based on the first reaction time, either paw-licking or jumping on a hot plate set at 55 °C with a cutoff time of 15 s. The time taken in seconds for the forepaw licking or jumping was taken as a latency period and was measured at regular time intervals of 0, 30, 60, 90, and 120 min (minutes). The percentage inhibition of pain was calculated for time points 30, 60, 90, and 120 min using the following equation, Equation (4):

(4) $$\mathrm{Inhibition}\;\mathrm{of}\;\mathrm{pain}\%=\left(\frac{P_{0}-P}{n-P\;}\right)\times 100$$

where *P* is the time taken by the animal to feel pain in the control group, *P*_0_ is the time taken by the animal to feel pain in the treatment groups, and *n* is the cutoff time.

### 3.10. Cytotoxicity Testing of the 95% Aqueous-Ethanolic Extract

#### 3.10.1. Cell Lines and Cell-Culturing

MDA, MCF-7, PANC-1, and normal human fibroblast cell lines were provided by Hamdi Mango Centre for Scientific Research, University of Jordan. All the cells were grown in a humidified 5% CO_2_ atmosphere incubated at 37 °C and in DMEM high glucose (Euroclone, S.p.A) containing 10% FBS (fetal bovine serum), 10 g/L penicillin-streptomycin, and 10 g/L l-glutamine.

#### 3.10.2. Extract Preparation for Cell-Line Proliferation Assay

Of *S. cyclophylla* 95% aqueous-ethanolic extract, 10 mg was dissolved in 500 µL of DMSO and raised to 1 mL with media to prepare the stock solution. Of this stock solution, 40 µL was diluted to 1 mL with media to prepare the first concentration of 400 μg/mL with 2× diluted to a final concentration of 6.25 μg/mL.

#### 3.10.3. Cell Proliferation Assays

To assess cell viability after incubation with various concentrations of *S. cyclophylla* 95% aqueous-ethanolic extract, MTT (3-(4,5-dimethyl-thiazol-2-yl)-2,5-diphenyltetrazolium bromide) assay (Promega, Madison, WI, USA) was used. The MTT assay depends on reducing MTT by mitochondrial dehydrogenase to the blue formazan material, which detects the mitochondria’s normal role and exhibits cell viability. The cells were suspended at a density of 5 × 10^4^ cells/mL in the media. Then 100 μL of each cell type were cultured into each well of 96-wells microtiter plates (5000 cells/well) and incubated for 24 h. The addition of *S. cyclophylla* 95% aqueous-ethanolic extract dissolved in DMSO to the wells was completed in triplicate to a final concentration range from 400 to 6.25 μg/mL in 2× serial dilutions and incubated at 37 °C, 5% CO_2_ for 24 h. The control wells without plant extracts (cells with DMSO or media) and negative control wells containing only fresh media were also used. The doxorubicin was used as a positive control. The MTT assay was performed according to the manufacturer’s instructions. Briefly, 20 μL of PBS containing 5 mg/mL MTT was added to each well of the plate, and then the plates were incubated for 3 h at 37 °C, MTT was carefully withdrawn, and 200 μL of DMSO was added to every well to dissolve the formed crystals. The absorbance at 570 nm and 630 nm read on a plate reader (BioteK Microplate Reader ELX800, Madison, WI, USA) was used. The difference in both the readings was used for the analysis of the results. The surviving cells’ percentage was calculated after normalization using GraphPad prism 6.01 software (GraphPad Software, San Diego, CA, USA).

### 3.11. Statistical Analyses

The data are expressed as the mean ± SEM. The differences among the groups for analgesic and anti-inflammatory activities were analyzed using a one-way analysis of variance (ANOVA). The two-way ANOVA for determinations of differences among the groups for the DPPH and iron chelation activities was followed by the posthoc test using Tukey’s multi-group comparison on GraphPad Prism 8.0.2. The effective concentration (IC_50_) was calculated using a nonlinear regression model on GraphPad Prism 8.0.2. The significance value was set at *p* < 0.05.

## 4. Conclusions

The current study provided confirmations of the traditionally claimed anti-inflammation and analgesic bioactivities of the halophytic herb. The frequent use of the *S. cyclophylla* aerial parts as animal-feed, emergency-food, salad, medicine, and nutraceutical was corroborated with the richness of the phytoconstituents, especially the flavonoids, and phenolics in the plant. The comparatively higher levels of the trace and essential elements in the plant, as compared to the non-halophytic plants, provided support to the exhibition of plant’s nutritional value, and pharmacological action of anti-inflammation and the analgesic bioactivity, which is also a factor of the phenolics, flavonoids, and other phytoconstituents presence in the plant. The cytotoxic and acute toxicity testing confirmed the safety of the plant’s use albeit under the limits of the tested dose, and to an extent of the use-period and dose frequency. Additionally, the molecular mechanistic details of these pharmacological actions, the role of trace elements, and detailed confirmations of the plant’s nutritional values need further study. The investigations on the chloroform, ethyl acetate, and aq.-ethanol extracts, especially the aq.-ethanol extract, holds promise for encountering new flavonoid(s) and phenolics-based structural entities of biological activity interest, which could also be a new molecule or a new molecular template of structural interest. The phytochemical investigations of the aq.-ethanol extract, together with the chloroform and ethyl acetate extracts, may lead to novel glycosylated flavonoid(s), and structures of polar phenolics nature, followed by probably, hereto unknown and novel alkaloid constituent(s) and other compounds from various other types of chemical classes, all towards finding new molecular templates for analgesic action, and anti-inflammation active non-steroidal structure-type drug discovery lead. The possibilities of finding new structural templates, also suitable for various anti-oxidant-based pharmacological activities of neurological disorders, are presumably waiting to be discovered.

Furthermore, these findings may extend and promote informed usage of the plant in medicine, food, and agriculture sectors and promote investigations and applications in complementary and alternative medicine and their usage in the other natural therapy modalities.

## Figures and Tables

**Table 1:** LC-MS analysis of the 95%aqueous-ethanolic extract of the *S. cyclophylla.*

Serial	^¶^ RT (min)	Obtained Mass (amu)	^¶¶^ Cal. Mass (amu)	Molecular Formula	Compound’s Identity
1	0.55	341.1070 [M − H]^+^	341.1083	C_12_H_22_O_11_	Trehalose
2	2.21	165.0533 [M − H]^+^	165.0551	C_9_H_10_O_3_	Phenyl lactic acid
3	2.85	163.0377 [M − H]^+^	163.0395	C_9_H_8_O_3_	4-Hydroxycinnamic acid *
4	2.95	353.0859 [M − H]^+^	353.0872	C_16_H_18_O_9_	Chlorogenic acid *
5	2.97	167.0716 [M − H]^+^	167.0708	C_9_H_12_O_3_	Homovanillyl alcohol
6	3.30	179.0725 [M − H]^+^	179.0344	C₉H₈O₄	Caffeic acid *
7	4.22	595.1668 [M + H]^+^	595.1663	C₂₇H₃₀O₁₅	Kaempferol-3-*O*-rutinoside
8	4.60	463.0879 [M + H]^+^	463.0876	C₂₁H₁₈O₁₂	Scutellarein-7-glucuronide
9	4.76	471.1875 [M − H]^+^	471.1866	C_22_H_32_O_11_	Eugenol rutinoside
10	4.78	183.0274 [M − H]^+^	183.0293	C_8_H_8_O_5_	4-*O*-Methyl gallic acid
11	4.83	503.3346 [M + H]^+^	503.3372	C₃₀H₄₆O₆	Medicagenic acid
12	4.89	463.0886 [M − H]^+^	463.0876	C_21_H_20_O_12_	Spiraeoside
13	4.90	611.1975 [M + H]^+^	611.1976	C₂₈H₃₄O₁₅	Hesperidin
14	4.95	447.0890 [M − H]^+^	447.0927	C_21_H_20_O_11_	Orientin
15	5.12	433.1099 [M − H]^+^	433.1134	C_21_H_22_O_10_	3-Glu-3,4’,7-trihydroxyisoflavanone
16	5.16	193.0551 [M − H]^+^	193.0500	C₁₀H₁₀O₄	Ferulic acid *
17	5.28	739.2096 [M − H]^+^	739.2085	C_33_H_40_O_19_	3-*O*-Neohesperidoside-7-rha kaempferol
18	5.54	593.1488 [M − H]^+^	593.1506	C_27_H_30_O_15_	3,7-Dirhamnosyl quercetin
19	5.60	609.1427 [M − H]^+^	609.1455	C_27_H_30_O_16_	3-Glu-7-rham quercetin
20	5.73	463.0861 [M − H]^+^	463.0876	C_21_H_20_O_12_	Hyperoside *
21	5.90	341.1392 [M + H]^+^	341.1389	C₂₀H₂₀O₅	8-Prenyl naringenin
22	5.91	328.1171 [M − H]^+^	328.1184	C_18_H_19_NO_5_	Hernandine
23	5.92	447.0906 [M − H]^+^	447.0927	C_21_H_20_O_11_	Luteolin-7-*O*-glucosid
24	6.32	577.1539 [M − H]^+^	577.1557	C_27_H_30_O_14_	Isorhoifolin
25	6.36	593.1472 [M − H]^+^	593.1506	C_27_H_30_O_15_	3-*O*-Neohesperidoside kaempferol
26	6.51	607.1670 [M − H]^+^	607.1663	C_28_H_32_O_15_	Diosmin
27	6.57	447.0918 [M − H]^+^	447.0927	C_21_H_20_O_11_	Kaempferol-3-*O*-glucoside
28	6.73	187.0948 [M − H]^+^	187.0970	C_9_H_16_O_4_	Azelaic acid (Nonandioic acid)
29	6.81	285.0402 [M − H]^+^	285.0399	C_15_H_10_O_6_	3,6,2’,4’-Tetrahydroxyflavone
30	6.81	447.0935 [M − H]^+^	447.0927	C_21_H_20_O_11_	Luteolin-4’-*O*-glucoside
31	6.90	431.0985 [M − H]^+^	431.0978	C_21_H_20_O_10_	Apigetrin
32	7.35	325.1416 [M + H]^+^	325.1439	C₂₀H₂₀O₄	Isobavachin
33	7.65	177.0546 [M + H]^+^	177.0551	C₁₀H₈O₃	4-Methyl umbelliferone
34	7.72	289.1097 [M − H]^+^	289.1076	C_16_H_18_O_5_	5-*O*-Methyl visamminol
35	8.06	312.1217 [M − H]^+^	312.1235	C_18_H_19_NO_4_	Acetyl caranine
36	8.51	181.0507 [M − H]^+^	181.0500	C_9_H_10_O_4_	Homovanillic acid
37	8.59	301.0335 [M − H]^+^	301.0348	C_15_H_10_O_7_	Quercetin *
38	8.69	437.1944 [M + H]^+^	437.1964	C₂₆H₂₈O₆	Artocarpin
39	8.80	433.1131 [M + H]^+^	433.1134	C₂₁H₂₀O₁₀	Genistin
40	9.24	315.0506 [M − H]^+^	315.0504	C_16_H_12_O_7_	6-Methoxy luteolin
41	10.35	315.0508 [M − H]^+^	315.0504	C_16_H_12_O_7_	Rhamnetin
42	11.70	329.2309 [M − H]^+^	329.2328	C_18_H_34_O_5_	Pinellic acid
43	11.96	247.1337 [M − H]^+^	247.1334	C_15_H_20_O_3_	3-Oxocostusic acid
44	12.71	359.1149 [M + H]^+^	359.1130	C₁₉H₁₈O₇	Corymbosin
45	13.68	283.0575 [M + H]^+^	283.0606	C₁₆H₁₀O₅	Pseudobaptigenin
46	14.25	293.1737 [M − H]^+^	293.1752	C_17_H_26_O_4_	6-Gingerol
47	14.25	285.1129 [M + H]^+^	285.1126	C₁₇H₁₆O₄	Caffeic acid phenethyl ester *
48	25.50	253.2149 [M − H]^+^	253.2167	C₁₆H₃₀O₂	Palmitoleic acid
49	25.68	255.2308 [M − H]^+^	255.2324	C_16_H_32_O_2_	Palmitic acid
50	27.91	279.2304 [M − H]^+^	279.2324	C_18_H_32_O_2_	Linoleic acid
51	28.23	281.2464 [M − H]^+^	281.2480	C_18_H_34_O_2_	Oleic acid
52	29.94	283.2620 [M − H]^+^	283.2637	C_18_H_36_O_2_	Octadecanoic acid

^¶^ RT Retention Time, ^¶¶^ Cal. Calculated, * Compounds identified by comparison with their respective standards, rest are tentatively identified. Supplementary File, Figure S1: LC-MS chromatogram.

**Table 2:** Trace elements and heavy metals in *S.* *cyclophylla.*

Element	Concentration
Magnesium (Mg)	2.90 g/kg
Iron (Fe)	172.60 mg/kg
Zinc (Zn)	49.66 mg/kg
Manganese (Mn)	9.95 mg/kg
Copper (Cu)	7.81 mg/kg
Lead (Pb)	319.00 µg/kg
Cadmium (Cd)	109.00 µg/kg

**Table 3:** Quantitative analyses of the total phenolics and flavonoids contents in *S. cyclophylla.*

Extracts	Total Phenolics *	Total Flavonoids **
*n*-Hexane	32.70 ± 0.01	1.68 ± 0.01
Chloroform	85.38 ± 0.04	5.38 ± 0.00
Ethyl acetate	32.40 ± 0.02	5.94 ± 0.04
Aqueous-Ethanol	136.08 ± 0.12	3.19 ± 0.03

* mg gallic acid equivalent/g of the dried extract, ** mg quercetin equivalent/g of the dried extract.

**Table 4:** Analgesic effects of the *S. cyclophylla* extracts, and% inhibition of pain in mice assessed by hot-plate method ^‡^.

Group	0 min	30 min	60 min	90 min	120 min
Olive oil	10.63 ± 1.28	12.33 ± 0.91	10.07 ± 0.61	11.27 ± 0.26	10.13 ± 0.75
Diclofenac % Inhibition	11.07 ± 0.53	12.43 ± 1.31	14.37 ± 0.40 *	14.73 ± 0.16 **	13.60 ± 0.88 *
		3.75%	87.16%	92.86%	71.23%
*n*-Hexane Ext., % Inhibition	7.60 ± 0.91	12.43 ± 0.18	12.43 ± 1.52	12.87 ± 0.52	13.57 ± 0.90 *
		3.75%	47.97%	42.86%	70.55%
Chloroform Ext., % Inhibition	8.87 ± 0.62	11.20 ± 1.23	12.80 ± 1.39	11.93 ± 1.04	13.60 ± 0.46 *
		−42.50%	55.41%	17.86%	71.23%
Ethyl acetate Ext., % Inhibition	9.72 ± 0.77	8.13 ± 0.67	12.07 ± 0.84	11.99 ± 0.48	12.87 ± 0.73
		−157.50%	40.54%	19.42%	56.16%
Aqueous-ethanolic Ext., % Inhibition	11.47 ± 1.22	11.50 ± 1.11	14.50 ± 0.31 *	14.53 ± 0.29 **	14.98 ± 0.01 ***
		−31.25%	89.86%	87.50%	99.66%

^‡^ Latency period values for all the measured groups at different points are denoted as mean ± SEM, *n* = 6 animals/group. The statistical significance via one-way ANOVA followed by a posthoc test using Tukey’s multi-group comparison: * *p* < 0.05, ** *p* < 0.01, and *** *p* < 0.001 compared to the olive oil group. Pain inhibition% is compared to the olive oil group at different time points (30, 60, 90, and 120 min). For example, at 120 min compared to the olive group, the inhibition was 71.23%, 99.66%, 70.55%, 56.16%, and 71.23% for the diclofenac aqueous-ethanolic, n-hexane, ethyl acetate, and chloroform extracts, respectively. Supplementary file, Table S2: Analgesic activity’s raw data.

**Table 5:** Inhibition of inflammation by *S. cyclophylla* extracts following carrageenan-induced paw edema in rats ^‡^.

Group	0 h	1 h	2 h	3 h	6 h	24 h
Olive oil, paw volume (mL)	1.03 ± 0.01	1.07 ± 0.01	1.52 ± 0.01	1.54 ± 0.02	1.54 ± 0.02	1.47 ± 0.01
Diclofenac, paw volume, % inhibition	0.97 ± 0.01	0.98 ± 0.01	1.01 ± 0.01 ****	1.10 ± 0.01 ****	1.18 ± 0.01 ****	1.29 ± 0.01 ***
		8.39%	33.60%	28.82%	23.23%	12.26%
*n*-Hexane Ext., paw volume, % inhibition	1.08 ± 0.00	1.20 ± 0.04 *	1.34 ± 0.02 ***	1.38 ± 0.03 ***	1.41 ± 0.03 *	1.17 ± 0.02 ****
		−11.92%	11.71%	10.37%	8.59%	20.44%
Chloroform Ext., paw volume, % inhibition	1.06 ± 0.01	1.05 ± 0.01	1.09 ± 0.02 ****	1.17 ± 0.04 ****	1.35 ± 0.07 ****	1.38 ± 0.06
		2.07%	28.18%	24.14%	12.48%	5.98%
Ethyl acetate Ext., paw volume,% inhibition	1.05 ± 0.01	1.20 ± 0.04 *	1.08 ± 0.04 ****	1.18 ± 0.04 ****	1.21 ± 0.07 ****	1.21 ± 0.03 ****
		−11.92%	28.99%	23.49%	21.43%	18.09%
Aqueous-Ethanolic Ext., paw volume, % inhibition	1.04 ± 0.01	0.89 ± 0.03 ***	0.89 ± 0.02 ****	1.01 ± 0.02 ****	1.16 ± 0.06 ****	1.33 ± 0.04 **
		16.79%	41.07%	34.51%	24.82%	9.39%

^‡^ Rat paw volume values for all the measured groups at different time-points are denoted as mean ± SEM, *n* = 6 animals/group. Statistical significance were performed via a one-way ANOVA followed by a posthoc test using Tukey’s multi-group comparison: * *p* < 0.05, ** *p* < 0.01, *** *p* < 0.001, and **** *p* < 0.0001 compared to the olive oil group for the measured time points from 1 to 24 h. Edema inhibition% as compared to the olive oil group at different time points from 1 to 24 h. For example, at 2 h, compared to the olive oil group, the inhibition% was at 33.6%, 41.1%, 11.7%, 29.0%, and 28.2% for the diclofenac aqueous-ethanolic, n-hexane, ethyl acetate, and chloroform extracts, respectively. Supplementary file, Table S1: Anti-inflammatory activity’s raw data.

## Data Availability

Data is contained within the article and in the Supplementary File.

## Supplementary Materials

The following are available online. Figure S1: LC-MS chromatogram, Table S1: Anti-inflammatory activity’s raw data, Table S2: Analgesic activity’s raw data, Table S3: Cytotoxic activity’s raw data.

## Sample Availability

Samples of the plant and its extracts are available from the authors.
