# Supplementary material for: Phytochemical Analysis, Pharmacological and Safety Evaluations of Halophytic Plant, Salsola cyclophylla
Source: Molecules. 2021 Apr 20;26(8):2384. doi: 10.3390/molecules26082384 (PMC8073378; doi:10.3390/molecules26082384)
Supplement: Supplementary file 1 [file molecules-26-02384-s001.zip › molecules-1167601-conversion-sm.pdf]

Figure S1: LC-MS chromatogram

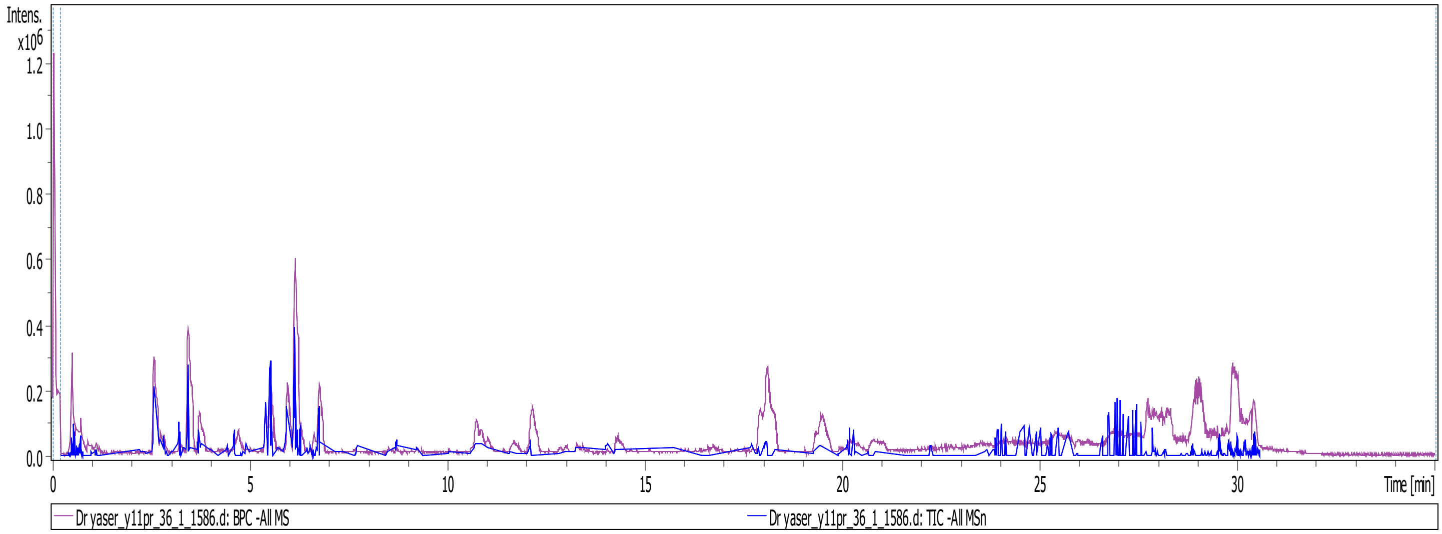

Table S1: Anti-inflammatory activity raw data

Olive Oil	0 Hour	1 Hour	2 Hour	3 Hour	6 Hour	24 Hour
Rat 1	1	1.06	1.55	1.62	1.6	1.49
Rat 2	1.02	1.05	1.47	1.46	1.48	1.42
Rat 3	1.07	1.11	1.53	1.55	1.54	1.49
Rat 4	1.03	1.07	1.52	1.54	1.54	1.47
Rat 5	1.04	1.08	1.53	1.55	1.55	1.48
Rat 6	1.02	1.06	1.51	1.53	1.53	1.46
**Average**	**1.03**	**1.07**	**1.52**	**1.54**	**1.54**	**1.47**
**S.D**	**0.02**	**0.02**	**0.03**	**0.05**	**0.04**	**0.03**
**n**	**6.00**	**6.00**	**6.00**	**6.00**	**6.00**	**6.00**
**S.E**	**0.01**	**0.01**	**0.01**	**0.02**	**0.02**	**0.01**
**Diclofenac**						
Rat 1	0.95	0.97	1	1.14	1.21	1.32
Rat 2	0.99	1.01	1.03	1.11	1.19	1.29
Rat 3	0.96	0.97	0.99	1.04	1.15	1.25
Rat 4	0.97	0.98	1.01	1.10	1.18	1.29
Rat 5	0.98	0.99	1.02	1.11	1.19	1.3
Rat 6	0.96	0.97	1	1.09	1.17	1.28
**Average**	**0.97**	**0.98**	**1.01**	**1.10**	**1.18**	**1.29**
**S.D**	**0.01**	**0.02**	**0.01**	**0.03**	**0.02**	**0.02**
**n**	**6.00**	**6.00**	**6.00**	**6.00**	**6.00**	**6.00**
**S.E**	**0.01**	**0.01**	**0.01**	**0.01**	**0.01**	**0.01**

Aq.-ethanol						
Rat 1	1.06	0.9	0.92	1.1	1.4	1.45
Rat 2	1.04	0.76	0.81	0.92	0.97	1.18

Rat 3	1.02	1.02	0.9	1.01	1.1	1.36
Rat 4	1.04	0.89	0.88	1.01	1.16	1.33
Rat 5	1.05	0.9	0.99	1.02	1.17	1.34
Rat 6	1.03	0.88	0.87	1	1.15	1.32
**Average**	**1.04**	**0.89**	**0.89**	**1.01**	**1.16**	**1.33**
**S.D**	**0.01**	**0.08**	**0.06**	**0.06**	**0.14**	**0.09**
**n**	**6.00**	**6.00**	**6.00**	**6.00**	**6.00**	**6.00**
**S.E**	**0.01**	**0.03**	**0.02**	**0.02**	**0.06**	**0.04**

**n-hexane**						
Rat 1	1.09	1.36	1.41	1.25	1.29	1.22
Rat 2	1.09	1.12	1.36	1.52	1.5	1.08
Rat 3	1.07	1.12	1.25	1.38	1.43	1.2
Rat 4	1.08	1.20	1.34	1.38	1.41	1.17
Rat 5	1.09	1.21	1.35	1.39	1.42	1.18
Rat 6	1.07	1.19	1.33	1.37	1.4	1.16
**Average**	**1.08**	**1.20**	**1.34**	**1.38**	**1.41**	**1.17**
**S.D**	**0.01**	**0.09**	**0.05**	**0.09**	**0.07**	**0.05**
**n**	**6.00**	**6.00**	**6.00**	**6.00**	**6.00**	**6.00**
**S.E**	**0.00**	**0.04**	**0.02**	**0.03**	**0.03**	**0.02**

**Ethyl acetate**						
Rat 1	0.99	1.07	0.98	1	0.98	1.24
Rat 2	1.07	1.14	0.99	1.24	1.15	1.07
Rat 3	1.08	1.39	1.26	1.3	1.5	1.3
Rat 4	1.05	1.20	1.08	1.18	1.21	1.20
Rat 5	1.06	1.21	1.09	1.19	1.22	1.21
Rat 6	1.04	1.19	1.07	1.17	1.2	1.19
**Average**	**1.05**	**1.20**	**1.08**	**1.18**	**1.21**	**1.20**
**S.D**	**0.03**	**0.11**	**0.10**	**0.10**	**0.17**	**0.08**
**n**	**6.00**	**6.00**	**6.00**	**6.00**	**6.00**	**6.00**
**S.E**	**0.01**	**0.04**	**0.04**	**0.04**	**0.07**	**0.03**

**Chloroform**						
Rat 1	1.02	1.07	1.18	1.35	1.57	1.57
Rat 2	1.08	1.01	1.01	1.08	1.07	1.15
Rat 3	1.07	1.07	1.08	1.08	1.4	1.42
Rat 4	1.06	1.05	1.09	1.17	1.35	1.38
Rat 5	1.07	1.06	1.1	1.18	1.36	1.39
Rat 6	1.05	1.04	1.08	1.16	1.34	1.37
**Average**	**1.06**	**1.05**	**1.09**	**1.17**	**1.35**	**1.38**
**S.D**	**0.02**	**0.02**	**0.05**	**0.10**	**0.16**	**0.13**
**n**	**6.00**	**6.00**	**6.00**	**6.00**	**6.00**	**6.00**
**S.E**	**0.01**	**0.01**	**0.02**	**0.04**	**0.07**	**0.06**

**Percentage inhibition of inflammation**						
CMC vs Diclofenac		**8.39**	**33.60**	**28.82**	**23.23**	**12.26**

CMC vs Ethanol		16.79	41.07	34.51	24.82	9.39
CMC vs n-hexane		-11.92	11.71	10.37	8.59	20.44
CMC vs Ethyl acetate		-11.92	28.99	23.49	21.43	18.09
CMC vs Chloroform		2.07	28.18	24.14	12.48	5.98

Table S2: Analgesic activity raw data

**Olive Oil**	**0 Min**	**30 Mins**	**60 Mins**	**90 Mins**	**120 Mins**
Mice 1	14.0	15.0	9.0	10.7	7.9
Mice 2	7.0	10.0	9.2	11.0	10.5
Mice 3	10.9	12.0	12.0	12.1	12.0
Mice 4	14.0	15.0	9.0	10.7	7.9
Mice 5	7.0	10.0	9.2	11.0	10.5
Mice 6	10.9	12.0	12.0	12.1	12.0
**Mean**	**10.63**	**12.33**	**10.07**	**11.27**	**10.13**
**SD**	**3.14**	**2.25**	**1.50**	**0.66**	**1.86**
**n**	**6.00**	**6.00**	**6.00**	**6.00**	**6.00**
**SEM**	**1.28**	**0.92**	**0.61**	**0.27**	**0.76**
**Diclofenac**					
Mice 1	9.4	8.3	13.1	14.2	10.8
Mice 2	12.1	14.0	15.0	15.0	15.0
Mice 3	11.7	15.0	15.0	15.0	15.0
Mice 4	9.4	8.3	13.1	14.2	10.8
Mice 5	12.1	14.0	15.0	15.0	15.0
Mice 6	11.7	15.0	15.0	15.0	15.0
**Mean**	**11.07**	**12.43**	**14.37**	**14.73**	**13.60**
**SD**	**1.30**	**3.23**	**0.98**	**0.41**	**2.17**
**n**	**6.00**	**6.00**	**6.00**	**6.00**	**6.00**
**SEM**	**0.53**	**1.32**	**0.40**	**0.17**	**0.89**
**Aq.-ethanol**					
Mice 1	11.1	10.0	13.5	13.6	15.0
Mice 2	8.3	9.5	15.0	15.0	15.0
Mice 3	15.0	15.0	15.0	15.0	15.0
Mice 4	11.1	10.0	13.5	13.6	15.0
Mice 5	8.3	9.5	15.0	15.0	15.0
Mice 6	15.0	15.0	15.0	15.0	14.9
**Mean**	**11.47**	**11.50**	**14.50**	**14.53**	**14.98**
**SD**	**3.01**	**2.72**	**0.77**	**0.72**	**0.04**
**n**	**6.00**	**6.00**	**6.00**	**6.00**	**6.00**
**SEM**	**1.23**	**1.11**	**0.32**	**0.30**	**0.02**
**n-hexane**					
Mice 1	7.4	13.0	7.6	10.6	10.7

Mice 2	10.2	12.3	14.7	13.6	15.0
Mice 3	5.2	12.0	15.0	13.7	15.0
Mice 4	7.4	13.0	7.6	12.0	10.7
Mice 5	10.2	12.3	14.7	13.6	15.0
Mice 6	5.2	12.0	15.0	13.7	15.0
**Mean**	**7.60**	**12.43**	**12.43**	**12.87**	**13.57**
**SD**	**2.24**	**0.46**	**3.75**	**1.29**	**2.22**
**n**	**6.00**	**6.00**	**6.00**	**6.00**	**6.00**
**SEM**	**0.92**	**0.19**	**1.53**	**0.53**	**0.91**
**Ethyl acetate**					
Mice 1	9.0	8.1	10.2	10.8	11.0
Mice 2	10.0	10.0	14.6	11.3	12.6
Mice 3	9.0	6.3	11.9	13.5	15.0
Mice 4	13.0	8.1	10.2	11.6	11.0
Mice 5	10.0	10.0	14.6	11.3	12.6
Mice 6	7.3	6.3	10.9	13.5	15.0
**Mean**	**9.72**	**8.13**	**12.07**	**11.99**	**12.87**
**SD**	**1.89**	**1.65**	**2.06**	**1.18**	**1.80**
**n**	**6.00**	**6.00**	**6.00**	**6.00**	**6.00**
**SEM**	**0.77**	**0.68**	**0.84**	**0.48**	**0.74**
**Chloroform**					
Mice 1	9.8	8.3	8.4	11.5	12.5
Mice 2	9.9	10.4	15.0	15.0	15.0
Mice 3	6.9	14.9	15.0	9.3	13.3
Mice 4	9.8	8.3	8.4	11.5	12.5
Mice 5	9.9	10.4	15.0	15.0	15.0
Mice 6	6.9	14.9	15.0	9.3	13.3
**Mean**	**8.87**	**11.20**	**12.80**	**11.93**	**13.60**
**SD**	**1.52**	**3.02**	**3.41**	**2.57**	**1.14**
**n**	**6.00**	**6.00**	**6.00**	**6.00**	**6.00**
**SEM**	**0.62**	**1.23**	**1.39**	**1.05**	**0.47**
**% inhibition of pain**					
CMC vs Diclofenac	**9.92**	**3.75**	**87.16**	**92.86**	**71.23**
CMC vs Ethanol	**19.08**	**-31.25**	**89.86**	**87.50**	**99.66**
CMC vs n-hexane	**-69.47**	**3.75**	**47.97**	**42.86**	**70.55**
CMC vs Ethyl acetate	**-20.99**	**-157.50**	**40.54**	**19.42**	**56.16**
CMC vs Chloroform	**-40.46**	**-42.50**	**55.41**	**17.86**	**71.23**

Table S3: Cytotoxic activity raw data

Conc. µg/ml	MDA			Average	S.D	n	S.E
400	95.29	96.79	89.72	**93.93**	**3.73**	**3**	**2.15**

200	97.64	97.64	99.57	**98.28**	**1.11**	**3**	**0.64**
100	98.07	116.27	99.79	**104.71**	**10.05**	**3**	**5.80**
50	105.78	91.01	101.28	**99.36**	**7.57**	**3**	**4.37**
25	112.63	114.99	113.49	**113.70**	**1.19**	**3**	**0.69**
12.5	115.42	104.71	110.92	**110.35**	**5.38**	**3**	**3.10**
6.25	101.07	101.93	105.57	**102.86**	**2.39**	**3**	**1.38**

	PANC-1						
400	65.22	67.89	77.26	**70.12**	**6.32**	**3**	**3.65**
200	97.32	119.06	99.33	**105.24**	**12.01**	**3**	**6.94**
100	118.73	111.04	128.43	**119.40**	**8.71**	**3**	**5.03**
50	109.7	138.46	112.37	**120.18**	**15.89**	**3**	**9.17**
25	101	109.03	102.34	**104.12**	**4.30**	**3**	**2.48**
12.5	122.07	132.11	131.77	**128.65**	**5.70**	**3**	**3.29**
6.25	107.36	106.76	124.08	**112.73**	**9.83**	**3**	**5.68**

	MCF7						
400	88.2	90.49	83	**87.23**	**3.84**	**3**	**2.22**
200	110.92	98.84	113.28	**107.68**	**7.75**	**3**	**4.47**
100	94.41	89.98	84.91	**89.77**	**4.75**	**3**	**2.74**
50	103	101.93	102.14	**102.36**	**0.57**	**3**	**0.33**
25	128.27	108.97	97.99	**111.74**	**15.33**	**3**	**8.85**
12.5	105.7	87.13	122.48	**105.10**	**17.68**	**3**	**10.21**
6.25	121.13	111.56	107.28	**113.32**	**7.09**	**3**	**4.09**

	Fibroblast						
400	64.24	64.03	69.16	**65.81**	**2.90**	**3**	**1.68**
200	84.15	91.01	96.79	**90.65**	**6.33**	**3**	**3.65**
100	87.37	85.44	89.51	**87.44**	**2.04**	**3**	**1.18**
50	105.35	100	90.58	**98.64**	**7.48**	**3**	**4.32**
25	100.64	105.35	113.49	**106.49**	**6.50**	**3**	**3.75**
12.5	116.49	119.49	108.78	**114.92**	**5.52**	**3**	**3.19**
6.25	114.56	104.07	113.06	**110.56**	**5.67**	**3**	**3.28**
